# Zoledronic acid blocks the interaction between breast cancer cells and regulatory T-cells

**DOI:** 10.1186/s12885-019-5379-9

**Published:** 2019-02-26

**Authors:** Hsien Liu, Shih-Han Wang, Shin-Cheh Chen, Ching-Ying Chen, Tsun-Mei Lin

**Affiliations:** 1 Department of Surgery, Chi Mei Medical Center, Liouying, Tainan, Taiwan; 2 Department of Chemical and Materials Engineering, National Yunlin University of Science and Technology, Yunlin, Taiwan; 3 Department of Surgery, Chang Gung Memorial Hospital, Taipei, Taiwan; 4 Department of Medical Laboratory Science, I-Shou University, Kaohsiung, Taiwan; 5 Departments of Medical Research, E-Da Hospital/I-Shou University, Kaohsiung, Taiwan

**Keywords:** Regulatory T-cells, Zoledronic acid, Breast cancer, Immunomodulation

## Abstract

**Background:**

Zoledronic acid (ZA), a nitrogen-containing bisphosphonate, inhibits osteoclastogenesis. Emerging evidence suggests that ZA has anti-tumor and anti-metastatic properties for breast cancer cells. In a mouse model of ZA-related osteonecrosis of the jaw, ZA administration was found to suppress regulatory T-cells (Tregs) function. Our previous reports also demonstrated ZA acted as an immune modulator to block Tregs. Manipulation of Tregs represents a new strategy for cancer treatment. However, the relationship among ZA, Tregs, and cancer cells remains unclear. In this study, we investigated the effects of ZA on the interaction of breast cancer cells and Tregs.

**Methods:**

The anti-tumor effect of ZA on triple negative breast cancer cell lines were validated by XTT, wound healing and apoptosis analysis. A flow cytometry-based assay was used to analyze the immunosuppressive effect of Tregs treated with media conditioned by breast cancer cells, and a transwell assay was used to evaluate the chemotactic migration of Tregs. Differential gene expression profile on MDA-MB-231 treated with ZA (25 μM) was analyzed by. microarrays to describe the molecular basis of actions of ZA for possible direct anti-tumor effects. Enzyme-linked immunosorbent assays and quantitative real-time PCR were used to investigate the effect of ZA on the expression of cytokines/factors by breast cancer cells.

**Results:**

ZA was found to inhibit the proliferation and migration of breast cancer cells. Media conditioned by the MDA-MB-231 cells promoted the expansion, chemotactic migration, and immunosuppressive activity of Tregs, and these effects were attenuated in a dose-dependent manner by ZA treatment, and the attenuation was due to reduced expression of selected breast cancer cell factors (CCL2, CCL5, and IDO).

**Conclusions:**

ZA can significantly affect the interaction between breast cancer cells and Tregs. Our findings indicate that ZA is a potential therapeutic agent that can be used to reduce cancer aggressiveness by abolishing the supportive role of Tregs.

**Electronic supplementary material:**

The online version of this article (10.1186/s12885-019-5379-9) contains supplementary material, which is available to authorized users.

## Background

Naturally occurring regulatory T-cells (Tregs, defined as CD4^+^CD25^+^FoxP3^+^ T-cells) play a critical role in suppressing CD4^+^CD25^−^ and CD8^+^ effector T-cell functions for modulation of immune responses. In addition, Tregs also play a significant role in the aggressiveness of cancer by suppressing tumor-specific immunity [1, 2]. The prevalence of Tregs has been demonstrated to increase in both the peripheral blood and tumor microenvironment of patients with invasive breast, pancreas, colon, or liver cancer [3, 4]. Evidence shows that certain cells with malignant phenotypes release chemokines and other substances, such as CCL5 (RANTES), CCL2 (MCP-1), CCL22, PGE2, and TGF-β, to attract and activate Tregs [5–10]. Tumor-infiltrating Tregs could promote local tumor growth and enhance tumor metastasis in the peripheral blood or lymphoid organs [11, 12]. Elucidating the factors responsible for trafficking and accumulation of Tregs in the tumor microenvironment and blocking the interaction between cancer cells and Tregs could offer attractive therapeutic targets for combating tumor-induced immune suppression [13, 14].

Zoledronic acid (ZA), a third-generation nitrogen-containing bisphosphonate, is the mainstay of treatment for bone disease associated with breast cancer [15]. ZA inhibits farnesyl diphosphate (FPP) synthase, the key enzyme of the mevalonate pathway, to block osteoclast-mediated bone resorption. Synthesis of FPP and geranylgeranyl diphosphate (which is required for the post-translational modification of small GTPases that regulate cell normal function synthesis) are blocked [16]. Apoptosis of osteoclasts is also induced by the production of triphosphoric acid 1-adenosine-5′-yl ester 3-[3-methylbut-3-enyl] ester (ApppI, cytotoxic ATP analogue) through FPP synthase inhibition. In addition, preclinical and clinical findings suggest that ZA might also have direct and indirect anti-tumor effects [17]. The effects of adjuvant ZA treatment on the overall survival of breast cancer patients were analyzed in several clinical trials. The trials revealed increased disease-free survival rates for patients who received an adjuvant therapy in combination with ZA in the ABCSG-12 trial and the ZO-FAST trial [18, 19]. However, a tendency for increased overall survival was identified in patients treated with an adjuvant therapy in combination with ZA compared with the adjuvant therapy alone in the AZURE trial [20]. Recent studies suggested that the anti-tumor and anti-metastatic properties of ZA might directly inhibit angiogenesis, tumor cell proliferation, and adhesion in bone and induce tumor cell apoptosis. In addition, the antitumor synergy of ZA with cytotoxic chemotherapy was also demonstrated to induce partial immunomodulatory effects through expansion of cytotoxic γδ T-cells and MHC-restricted αβ CD8+ T-cells, thus attenuating the macrophage-induced invasiveness of cancer cells and interfering with dendritic cell differentiation and maturation [21–23]. Although inhibition of Tregs activity has been demonstrated in the mouse model of ZA-related osteonecrosis of the jaw [24], the relationship among ZA, Tregs, and cancer cells is not yet clearly understood. In the current study, we investigated the possible vicious cycle of pro-malignant interaction between Tregs and breast cancer cells and the effect of ZA on this relationship. Our specific aims were to clarify the ability and expression of factors of breast cancer cells to attract and activate Tregs and to investigate the modulating effect of ZA on the ability of breast cancer cells to attract and activate Tregs.

## Methods

### Chemicals, antibodies, and cell line

ZA was sourced from Novartis Pharma AG Basel, Switzerland. Monoclonal antibodies against human CD25-PE were purchased from eBioscience (San Diego, CA, USA), CD69-FITC was sourced from BD Biosciences (San Jose, CA, USA); the secondary antibody goat anti-mouse IgG FITC was sourced from Merck Millipore (Billerica, MA); and human CCL2/MCP1, human CCL5/RANTES, anti-CCL2 antibody, and anti-CCL5 antibody were sourced from R & D Systems (Minneapolis MN). The triple negative breast cancer cell lines MDA-MB-231 and MDA-MB-468 were purchased from Food Industry Research and Development Institute (FIRDI) and Culture Collection and Research Center (CCRC, Taiwan, ROC). Both cell line were cultured with DMEM medium (Gibco®, Grand Island, NY, USA) containing 10% fetal bovine serum (FBS; Biological Industries, Kibbutz Beit HaEmek, Israel).

### Breast cancer cell proliferation assay

MDA-MB-231 breast cancer cells were seeded overnight in 96-well plates at 5 × 10^3^ cells/well, treated with various concentrations of ZA for 72 h, and assayed for cell viability using the XTT-cell proliferation kit (Biological Industries, Beit Haemek, Israel) during which the medium was replaced with fresh medium and XTT was added for 4 h. The absorbance of the samples was measured against a background control (used as a blank) in an ELISA reader set to a wavelength of 470 nm.

### Migration assays of breast cancer cells (wound healing assay)

MDA-MB-231 cells were grown to confluence in 6-cm dishes. Subsequently, the growth medium was removed, and the cell monolayers were ‘scratched’ with a 200-μl pipette tip. The MDA-MB-231 cells were further incubated in medium containing 0, 5, 10, or 25 μM ZA for 12 and 24 h and viewed under an inverted phase-contrast microscope (Zeiss, Primovert, Germany) to measure the cell migration distance.

### Apoptosis assays of breast cancer cells

MDA-MB-231 breast cancer cells were seeded at a density of 5 × 10^5^ cells/well, starved overnight in serum-free medium, treated with ZA (0, 10, 25 and 100 μM) for 48 h at 37 °C in DMEM with 2% FBS, washed with PBS, and incubated with FITC-labeled Annexin V and Propidium Iodide Staining Solution (eBioscience, San Diego, CA, USA). Samples were gently vortexed, incubated for 15 min in the dark, and analyzed within 1 h via flow cytometry using a FACScan flow cytometer (FACScalibur; BD Biosciences, San Jose, CA, USA) to determine percentages of apoptotic and necrotic cells.

### Preparation of breast cancer conditioned media (C.M)

Conditioned media (10% FBS C.M.) were harvested from MDA-MB-231 cells that had been starved overnight in serum-free DMEM and treated with 0, 10, and 25 μM ZA for 6 h. The cells were washed twice with phosphate buffered saline and cultured for 24 h in 10% FBS DMEM. All C.M. was collected, sterile filtered, and stored in aliquots at − 80 °C.

For chemo-attractive assay for Tregs, MDA-MB-231 cells were seeded overnight in 6-well plates at 5 × 10^5^ cells/well. Conditioned media (2% FBS C.M.) were harvested from MDA-MB-231 cells treated with 0, 10, and 25 μM ZA for 48 h.

### Isolation and expansion of regulatory T-cells

Tregs were immediately purified from peripheral blood mononuclear cells, which were isolated from 100 ml of fresh heparinized peripheral blood collected from healthy volunteers by Ficoll-Hypaque (GE Healthcare, Uppsala, Sweden) gradient centrifugation and by immunomagnetic separation using the Dynabeads® Regulatory CD4^+^CD25^+^ T-cell Kit (Invitrogen™, Oslo, Norway), according to the manufacturer’s instructions. The procedure yielded a highly pure preparation (> 90% purity) of regulatory CD4^+^CD25^+^ T-cells, more than 80% of which expressed the intracellular transcription factor Foxp3. The isolated Tregs were expanded with Dynabeads® Human Treg Expander (Gibco®, Oslo, Norway) containing 100 U/ml recombinant human interleukin (rIL-2) (Gibco®, Carlsbad, CA, USA). Each batch Tregs was treated with a standard protocol. After isolation, they were cultured for 10 days and expanded to more than 2 × 10^6^. Each experiment was performed by three independent batches in duplicate. The study was approved by the Institutional Review Committee of E-DA Hospital, and volunteer donors provided written informed consent.

### Regulatory T-cell viability/proliferation assay

To investigate the effect of breast cancer cell C.M. on Tregs viability/proliferation, Tregs were seeded in 24-well plates at 4 × 10^5^ cells/well, cultured with pure Tregs culture medium or a mixture of half Tregs culture medium and C.M. from MDA-MB-231 (10% FBS) with 6-h ZA (0, 10, 25 μM) pre-treatment, and stimulated with CD3/CD28 microbeads and rIL-2. The culture media were changed every 3 days, and the number of viable Tregs was counted using the trypan blue exclusion test.

### Migration assay of regulatory T-cells

Migration assays were performed in 24-well transwell chambers (Corning, New York, NY) using 8-μm pore polycarbonate filters. Expanded Tregs were added to the top chamber in serum-free RPMI medium at 5 × 10^4^ cells/100 μl. Various chemo-attractants, including 2% serum-containing DMEM with ZA (0, 10, and 25 μM) or 2% FBS C.M. of MDA-MB-231 cells pretreated with ZA (0, 10, and 25 μM) were added to the bottom chamber of the transwells in a volume of 650 μL. In certain experiments, the C.M. was preincubated with anti-CCL2 antibody (10 μg/ml), anti-CCL5 antibody (20 μg/ml), or both for 1 h. The migrated cells in the lower chamber were counted after incubation for 2 h at 37 °C. The chemotaxis index was calculated relative to the value obtained in response to 2% FBS-containing DMEM medium only.

### Immunosuppressive function assay of regulatory T-cells

The immunosuppressive activity of Tregs was analyzed with a Human Regulatory T-cell Function Kit (BD Biosciences, San Jose, CA, USA). Tregs (2 × 10^5^) were cultured with pure Tregs culture medium or half C.M. of ZA (0, 10, and 25 μM)-pretreated MDA-MB-231 cells and co-cultured with the responding effector T cells in PBMC (4 × 10^5^) stimulated using anti-CD3/CD28 beads to express activation marker CD69. After 7 h of activation, the percentage of CD69-positive effector T-cells was determined by flow cytometry, and the reduced expression of CD69 in the presence of Tregs indicated Treg suppressive capacity. The percent suppression was calculated using the following formula: 100 – [(% CD69-positive in the presence of Tregs/% CD69-positive in the absence of Tregs) × 100].

### Microarray gene expression profiling

Total cellular RNA was isolated from MDA-MB-231 cells treated with 25 μM ZA and with or without 100 ng/ml IFN-γ for 24 h by TRIzol (Invitrogen, Carlsbad, CA, USA) according to the manufacture’s protocol, and the total amount was quantified by measuring the optical density at 260 nm. Five micrograms of total RNA was reverse transcribed using the high capacity cDNA archive kit (Applied Biosystems, Foster City, CA, USA) according to vendor’s instructions. Human OneArray Plus (Phalanx Biotech, Hsinchu, Taiwan) was used for comparing gene expression profiles between cells treated with ZA and control. Labeling of cDNA, hybridization of labeled cDNA to genome probes, and scanning were performed according to the manufacturer’s instructions. Normalized spot intensities were transformed to gene expression log2 ratios between the control and ZA-treatment groups. The spots with log2 ratio ≥ 1 or log2 ratio ≤ − 1 and *p*-value < 0.05 were analyzed.

### Quantitative real-time polymerase chain reaction assay

The kynurenine pathway of tryptophan metabolism is as a key metabolic pathway contributing to immune escape for breast cancer cells [14]. Indoleamine 1,2 dioxygenase enzyme (IDO) activity was induced by IFN-γ [25] to reduce T-lymphocyte proliferation and to increase Tregs subpopulation expansion [22, 26]. In addition, chemokines released by tumor cells lead Tregs recruitment [27–29]. Therefore, we proposed that the modulating effect of ZA on breast cancer cells to decrease Tregs expansion, migration and activation.

Total RNA was extracted from MDA-MB-231 and MDA-MB-468 cells treated with ZA (0, 5, 10, and 25 μM) and with or without 100 ng/ml IFN-γ for 24 h using a Total RNA Mini Kit (Viogene, Sunnyvale, CA, USA). The first strand of cDNA was synthesized by reverse transcribing 1–2.5 μg of RNA with an iScriptTM cDNA Synthesis Kit (Bio-Rad, Foster City, CA, USA) according to the manufacturer’s instructions. Real-time PCR was performed using iTaqTM Universal SYBR@ Green Supermix (Bio-Rad, Foster City, CA, USA) with primers specific for the human gene (Table 1) and an ABI PRISM 7700 Sequence Detection System (Applied Biosystems, Warrington, WA, USA), according to the manufacturer recommendations, including performance of all reactions with three biological and negative controls. The CT is the threshold cycle number (i.e., the minimum number of cycles needed for PCR product detection). Analyses of relative gene expression were performed using the 2-ΔΔCT method, including normalization to the mRNA level of the housekeeping gene GADPH.

**Table 1:** Primer sequences used for qRT-PCR

Target gene	Oligonucleotide sequences (5′-3′)	
	Forward	Reverse
CCL2	CCC CAG TCA CCT GCT GTT AT	AGA TCT CCT TGG CCA CAA TG
CCL5	TGC CCA CGT CAA GGA GTA TTT C	AAC CCA CTT CTT CTC TGG GTT G
IDO	CAT CTG CAA ATC GTG ACT AAG	CAG TCG ACA CAT TAA CCT TCC TTC
GAPDH	TGA ACG GGA AGC TCA CTG G	TCC ACC ACC CTG TTG CTG TA

### Enzyme-linked immunosorbent assays for chemokines

MDA-MB-231 cells were seeded overnight in 6-well plates at 5 × 10^5^ cells/well, starved overnight at 37 °C in serum-free DMEM, and treated with ZA (0, 5, 10, and 25 μM) and 100 ng/ml IFN-γ for 48 h at 37 °C in 2% FBS-containing DMEM. CCL2 and CCL5 were detected in the supernatants of stimulated MDA-MB-231 cells using ELISA reagents obtained from R&D Systems. All samples were assayed in duplicate.

### Statistical analysis

The statistical analysis was performed using Prism version 5.00 software (GraphPad Software, USA). Data are presented as the mean ± SD from three independent experiments. All differences were tested for statistical significance between two groups with the two-tailed Student *t*-test, and for more than three groups, one-way ANOVA was applied. *P*-values of < 0.05 were considered statistically significant.

## Results

### ZA inhibits the proliferation and migration of MDA-MB-231 breast cancer cells

**Fig. 1:**
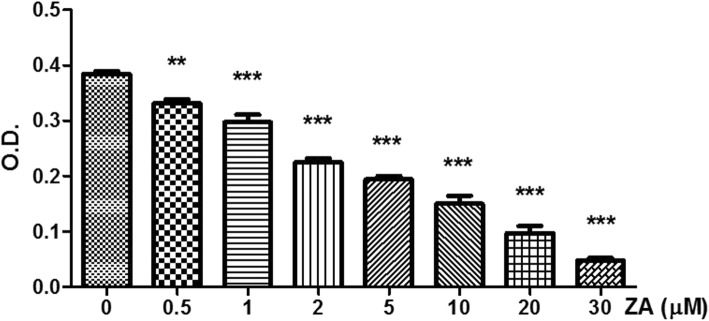
Effects of ZA treatment on proliferation of MDA-MB-231 cells. MDA-MB-231 cells were treated with various concentrations ZA for 72 h. Cell proliferation was determined by the XTT assay. Each data point represents the mean ± standard deviation based on quadruplicate determinations in three to five independent experiments. Significant difference was determined using one-way ANOVA; **P < 0.01, *** *P* < 0.001

**Fig. 2:**
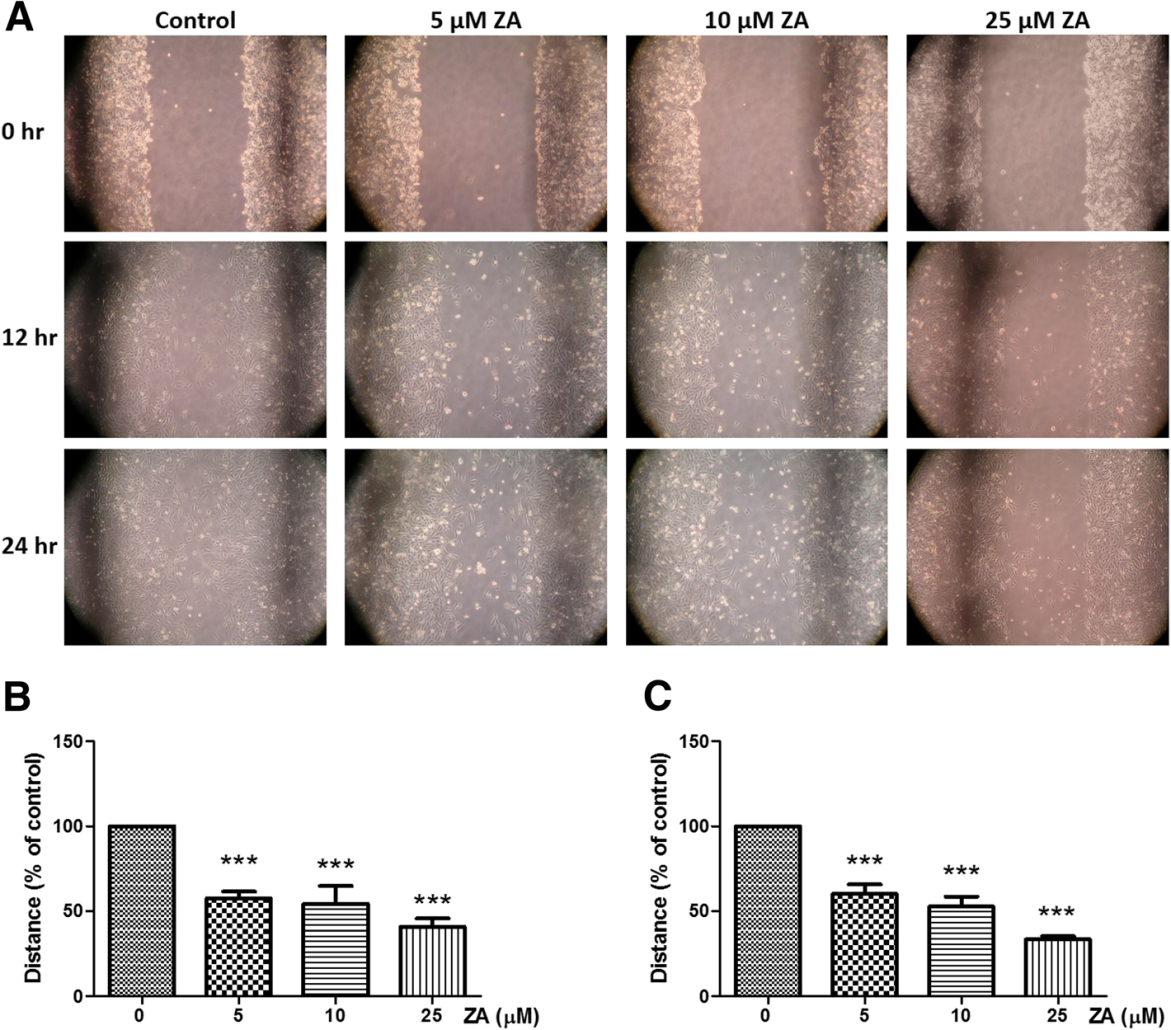
Effects of ZA treatment on migration of MDA-MB-231 cells. **a** Representative pictures of migration (wound closing) of MDA-MB-231 cells grown in the presence of various concentrations of ZA at 0, 12 and 24 h after wounding (100x magnifications). Quantification of migration distance as a percentage of the control value (ZA absent) in graphs of % wound closure after (**b**) 12 h and (**c**) 24 h of migration. The statistical analysis was performed using Prism version 5.00 software (GraphPad Software, USA). Data were analyzed using one-way ANOVA; *** *P* < 0.001

**Fig. 3:**
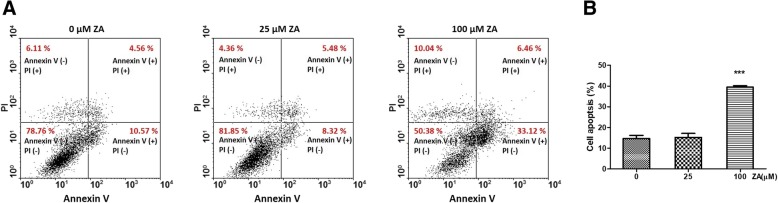
Effects of ZA treatment on apoptosis detected by annexin V/PI ataining. **a** The MDA-MB-231 cells were treated without or with various concentration of ZA for 48 h. Annexin V-FITC/PI staining was carried out and determined using flow cytometry. **b** The distribution of MDA-MB-231 cells undergoing early (annexin V+ PI-) and late apoptosis (annexin V+ PI+) were qualified, after treatment with ZA

As determined by XTT assay, ZA obviously inhibited the proliferation of MDA-MB-231 cells even at low concentrations (Fig. 1), which is consistent with previous reports [30]. The effect of ZA treatment on the migration of MDA-MB-231 cells was determined by the wound-healing assay. The rate of MDA-MB-231 cell migration was significantly decreased after ZA treatment for both 12 and 24 h (Fig. 2). Only 100 μM ZA increased induction of MDA-MB-231 cells apoptosis significantly, however, no increase of MDA-MB-231 cell apoptosis at concentrations of ZA under 25 μM (Fig. 3a & b). Therefore, concentration of 10 and 25 μM were used to treat MDA-MB-231 cells in vitro study.

### C. M. of ZA-pretreated MDA-MB-231 cells inhibit the proliferation of regulatory T-cells

**Fig. 4:**
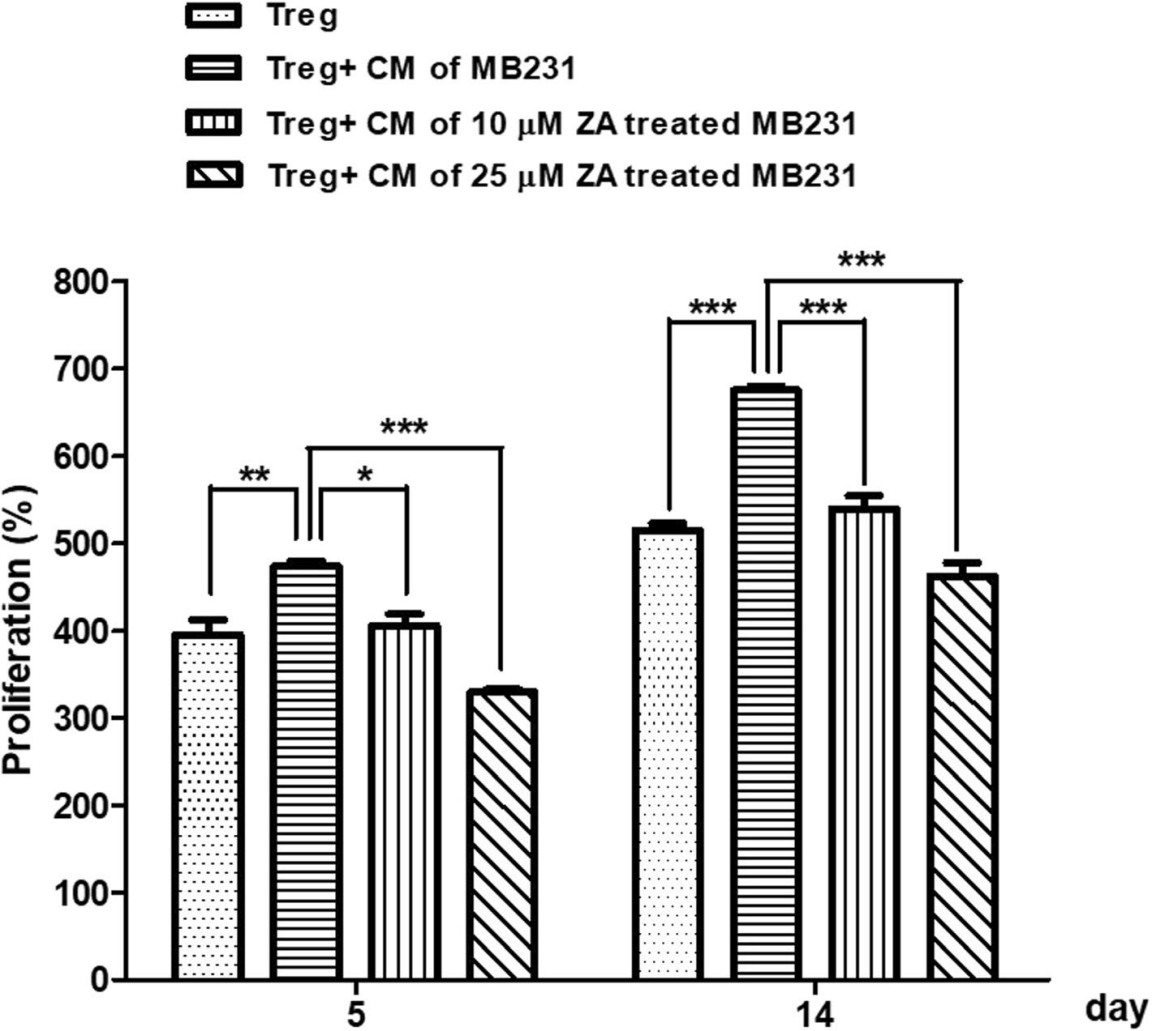
Effects of C.M. from ZA-pretreated MDA-MB-231 cells on Tregs proliferation. Tregs were cultured in the presence of 100 U/ml rIL-2 and Dynabeads® Human Treg Expander with or without C.M from ZA-pretreated MDA-MB-231 stimulation. Proliferation was expressed as the percentage of cell numbers relative to that at day 1 (100%). Proliferation percentages were calculated at day 5 and 14. Data are representative of three independent experiments. One-way ANOVA was applied to analyze the results. * *P* < 0.05, ** *P* < 0.01, *** *P* < 0.001

To examine the effects of C.M. of MDA-MB-231 cells on Tregs proliferation, we exposed Tregs to C.M. from ZA-pretreated MDA-MB-231 cells for 5 and 14 days and calculated the total numbers of viable Tregs in the presence or absence of C.M. from MDA-MB-231 cells. Exposure to C.M. from MDA-MB-231 cells (compared with mock C.M.) significantly amplified Tregs expansion by approximately 30–40%, and the effect was found as early as day 3–4 of incubation (*P* < 0.05) (Additional file 1: Figure S1). However, the enhancement was significantly blunted when MDA-MB-231 cells were pre-treated with ZA (Fig. 4). ZA pre-treatment attenuated the effect in a dose-dependent manner, with 10 μM ZA responsible for 20% growth inhibition (*p* < 0.001) and 25 μM ZA for 30% inhibition on the 14th day (*p* < 0.001).

### C.M. of ZA-pretreated MDA-MB-231 cells inhibit the migration of regulatory T-cells

**Fig. 5:**
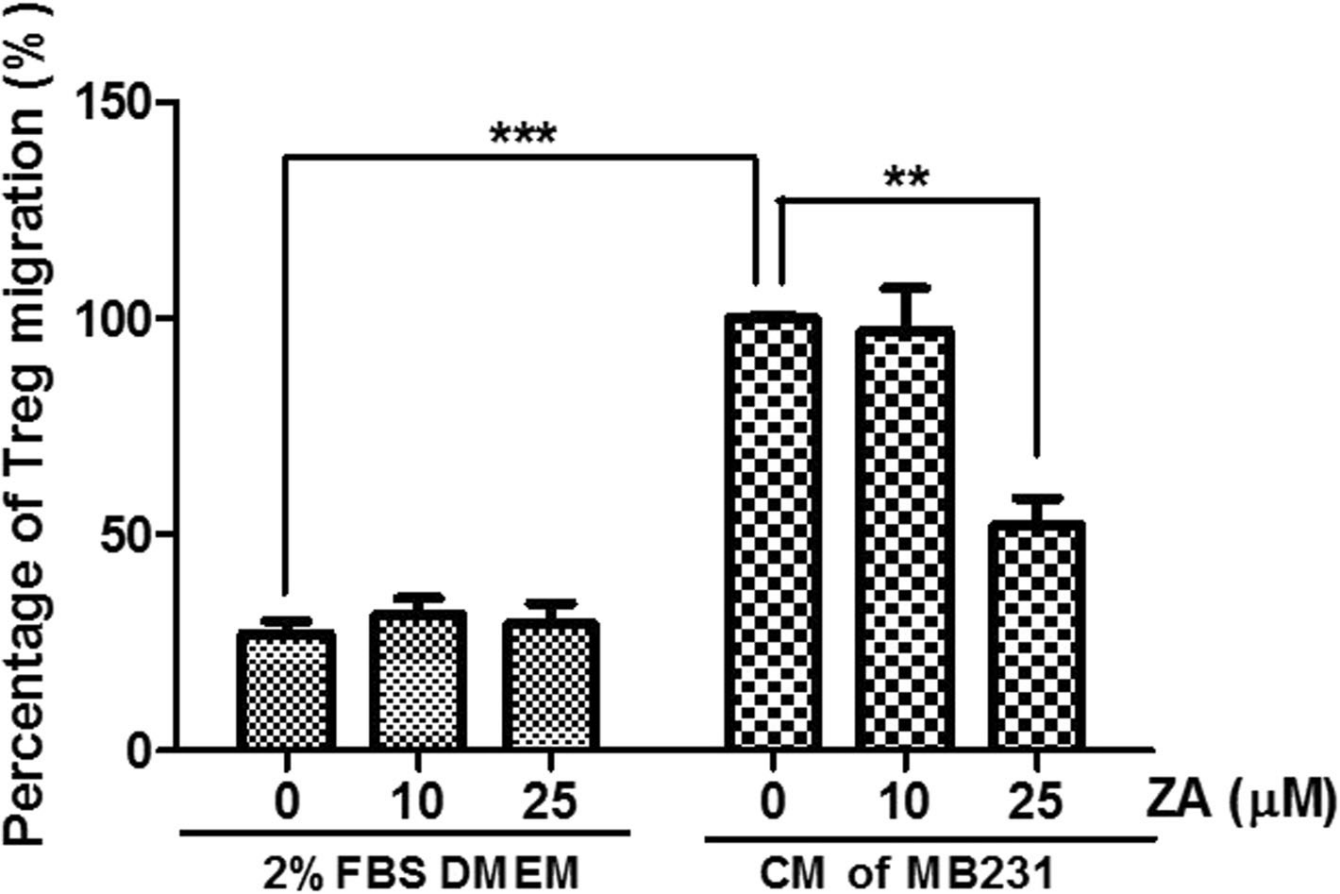
Inhibiting effects of C.M. from ZA-pretreated MDA-MB-231 cells on Tregs migration. Tregs (5 × 10 ^4^) were placed in the upper chambers for measurement of migration of Tregs into the lower chambers containing DMEM with 2% FBS with and without ZA and C.M. of MDA-MB-231 cells with and without ZA pretreatment. The relative percentages of migrated cells are shown compared with that of C.M. of MDA-MB-231 cells without ZA. Values indicate means ± SEM of results from three independent experiments performed in duplicate. Significant differences in percentage were determined using one-way ANOZA; ** *P* < 0.01; *** *P* < 0.001

We investigated whether ZA treatment affected the ability of breast cancer cells to attract Tregs. The results of transwell assays for Tregs migration through a polycarbonate membrane in response to chemoattractants showed that the chemoattractant activity of C.M. from MDA-MB-231 cells incubated for 48 h was 4 times that of 2% FBS medium only (*p* < 0.001) (Fig. 5). However, ZA had no direct effect on Tregs migration, only ZA pre-treated C.M. from MDA-MB-231 cells caused a significant dose-dependent reduction in the ability to attract Tregs migration (Fig. 5). C.M. from 25 μM ZA pretreated MDA-MB-231 cells decreased the chemotactic activity by 45% (*p* < 0.01).

### C.M. of ZA-pretreated MDA-MB-231 cells inhibit the immunosuppressive function of regulatory T-cells

**Fig. 6:**
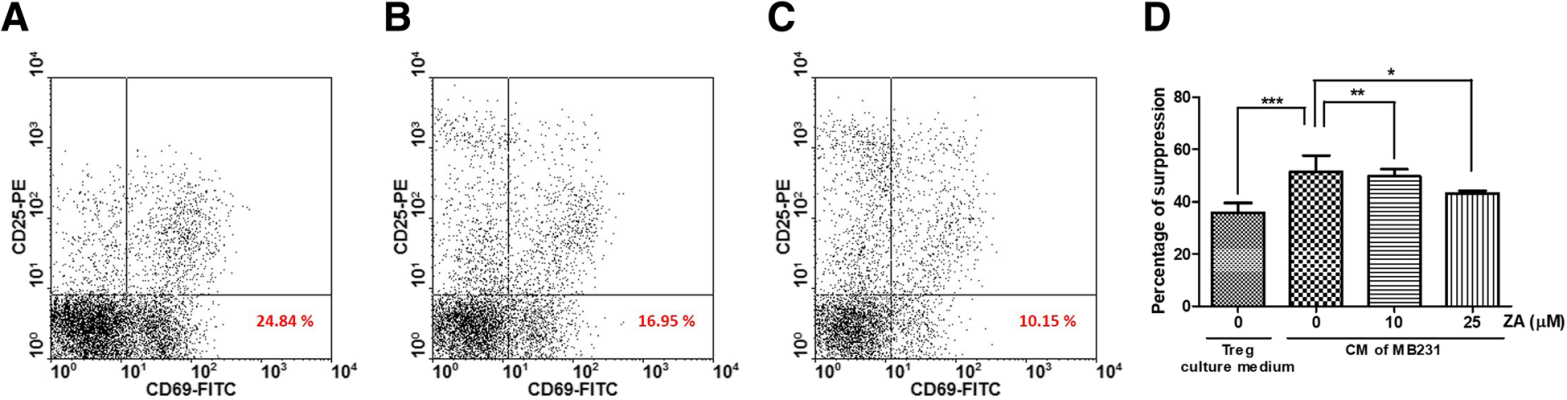
Inhibition effects of C.M. from ZA-pretreated MDA-MB-231 cells on Tregs immunosuppression function. **a** The percentage of responding effector T cells expressing activation marker CD69 in PBMC stimulated with anti-CD3/CD28 beads. **b** Tregs suppressed the percentage of responding effector T cells expressing activation marker CD69 in PBMC stimulated with anti-CD3/CD28 beads. **c** Tregs were cultured with C.M. of MDA-MB-231 cells and enhance the immunosuppression of Tregs to decrease responding effector T cells expressing activation marker CD69 in PBMC stimulated with anti-CD3/CD28 beads. **d** The percentage of immunosuppression was calculated using the following formulas: 100 – [(% CD69-positive in the presence of Tregs/% CD69-positive in the absence of Tregs) × 100]. Values shown are means ± SEM of results from three independent experiments. Significant differences were compared using one-way ANOVA; ** *P* < 0.01

We further investigated whether ZA treatment can affect the ability of MDA-MB-231 cells to activate the immunosuppressive activity of Tregs. Using a flow cytometry-based assay and the BD FastImmune Human Regulatory T-cell Function Kit, activation marker CD69 on the effector T-cell surface was analyzed. It was showed Treg celsl presence could suppressed the percentage of CD69 expression on effect T cells (Fig. 6b vs. a) and C.M. from MDA-MB-231 cells significantly activated the immunosuppressive function of Tregs (*p* < 0.001) (Fig. 6c vs. b). However, C.M. from MDA-MB-231 cells pre-treated with ZA significantly blunted this enhanced immunosuppressive activity in a dose-dependent manner (*p* < 0.01) (Fig. 6d).

### Gene expression profile of breast cancer cells treated with ZA

**Fig. 7:**
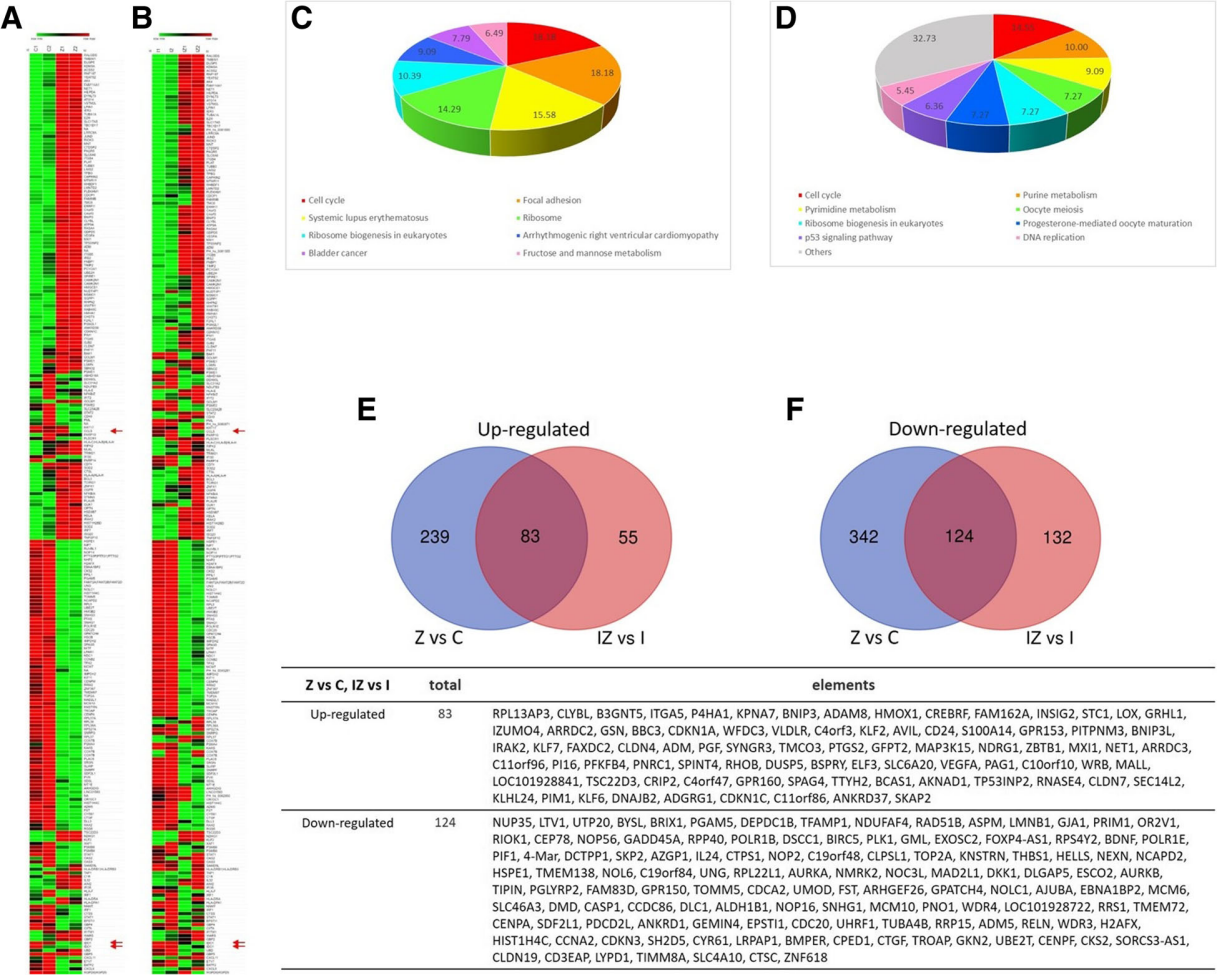
Treatment with ZA affects gene expression profile in MDA-MB-231cells and IFN-γ activated MDA-MB-231cells. **a**-**b** The human global gene expression profiles of two independent RNA samples were analyzed by human oligonucleotide DNA microarray at 48 h post-transfection. **c** Corrected microarray signal values of genes involved in different biological process, clustered by Biological process of MDA-MB-231 cells treated for 24 h with 25 μM ZA in comparison to control cells. **d** Corrected microarray signal values of genes involved in different biological process, clustered by Biological process of IFN-γ activated MDA-MB-231 cells treated for 24 h with 25 μM ZA in comparison to control cells. **e** Of the genes commonly down-regulated by ZA in MDA-MB-231cells and IFN-γ activated MDA-MB-231cells. **f** Of the genes commonly down-regulated by ZA in MDA-MB-231cells and IFN-γ activated MDA-MB-231cells

To investigate the molecular mechanisms by ZA exert their antitumor effects in MDA-MB-231 cells. We evaluated the expression profiling of MDB-MA-231 treated with 25 μM of ZA for 24 h versus untreated, using a cDNA microarray platform Affymetrix. Of the 33,000 independent features on the microarrays, 848 genes were found to be differentially expressed after 24 h of treatment (Fig. 7a). If MDB-MA-231 activated by 100 ng/ml IFN-γ and treated with 25 μM of ZA for 24 h, 421genes were found to be differentially expressed (Fig. 7b). We grouped genes related to molecular function categories that have changed in a statistically significant manner (*p*-value < 0.05) after treatment with ZA (Fig. 7c, d). The most significant changes in biological processes confirmed the involvement of ZA in cell cycle and focal adhesion. In particular, 86 genes were co-upregulated, and 128 genes were co-downregulated in ZA treatment either unstimulated or IFN-γ activated MDB-MA-231 (Fig. 7e, f).

### ZA treatment alters IDO and chemokines expression in breast cancer cell lines

**Fig. 8:**
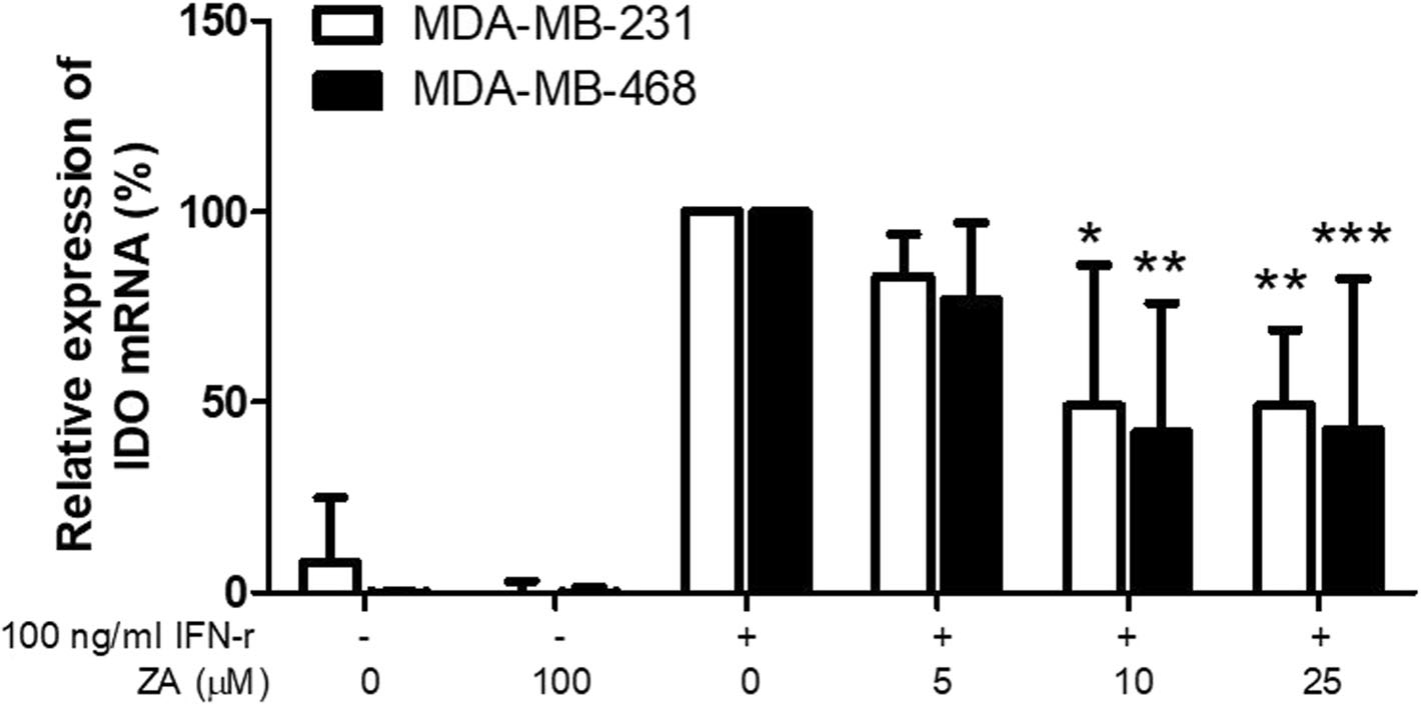
Influence of ZA on IDO mRNA expression by MDA-MB-231 and MDA-MB-468 cells. The mRNA levels of IDO were evaluated by qRT-PCR in 100 ng/ml IFN-γ  activated MDA-MB-231 and MDA-MB-468 cells treated with 0, 5, 10 and 25 μM ZA for 24 h. Gene expression values were normalized to GAPDH expression. Relative mRNA expression of IDO was calculated from cell treatment with ZA compared with cells only with IFN-γ stimulation. Values indicate means ± SEM of results from three independent experiments performed in duplicate (*n* = 3). * *P* < 0.05, *** *P* < 0.001

Using qRT-PCR, we analyzed MDA-MB-231 and MDA-MB-468 cells treated with various concentrations of ZA to determine their expression of IDO or chemokins that affect Tregs function. However, the mRNA and protein expression of IDO and chemokines (CCL2 and CCL5) by MDA-MB-231 and MDA-MB-468 cells were very low for detection. IDO activity could be induced by IFN-γ and increase Tregs subpopulation expansion [22, 26]. Therefore, the effects of ZA on IFN-γ treated triple negative breast cancer cell lines were analyzed. The results demonstrated IDO expression were significantly increased when the cells stimulated with IFN-γ but reduced in a dose-dependent manner by ZA treatment (Fig. 8). Collectively, ZA partially inhibited triple negative breast cancer cell-mediated enhancement of Tregs expansion through down-regulating IDO activity and via inhibition of the kynurenine/IDO axis.

**Fig. 9:**
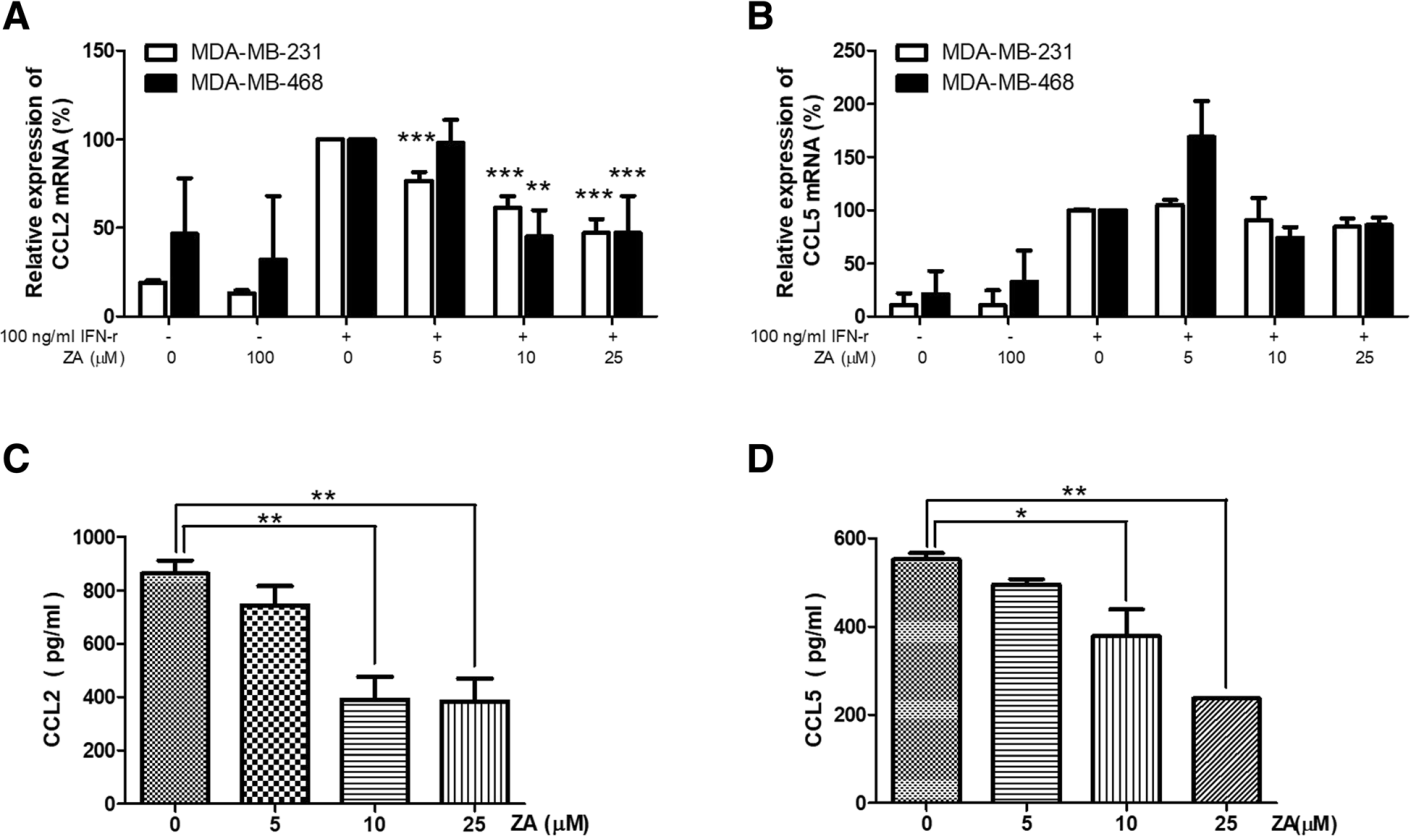
Chemokines expression in triple negative breast cancer cells treated with ZA. Relative mRNA levels of (**a**) CCL2 and (**b**) CCL5 were evaluated by qRT-PCR in MDA-MB-231 and MDA-MB-468 cells with or without 100 ng/ml IFN-γ activated in the presence of ZA (0, 5, 10 and 25 μM) for 24 h. Gene expression values were normalized to GAPDH expression. Concentrations of (**c**) CCL2 and (**d**) CCL5 were measured in the C.M. of 100 ng/ml IFN-γ treated MDA-MB-231 cells in the presence of ZA (0, 5, 10 and 25 μM) for 48 h by ELISA. Data were analyzed using one-way ANOVA; * *P* < 0.05, ** *P* < 0.01, *** *P* < 0.001

**Fig. 10:**
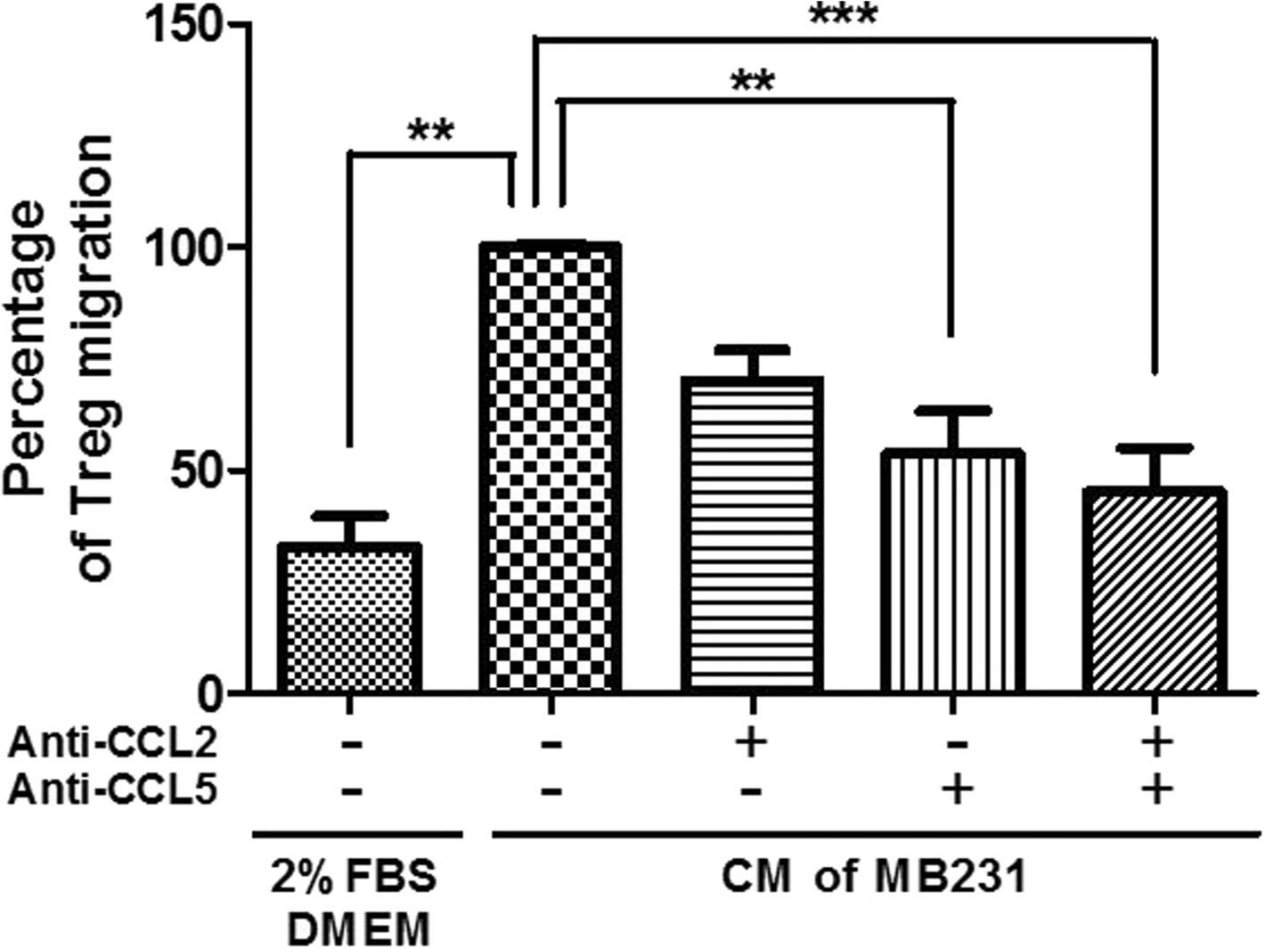
Inhibition of C.M. of MDA-MB-231 induced migration of Tregs following treatment with monoclonal antibody of CCL2 or CCL5. C.M. with or without addition of anti-CCL2 antibody (10 μg/ml), anti-CCL5 antibody (20 μg/ml), alone or in combination on the migration of Tregs was applied. The relative percentages of migrated cells were compared with that of C.M. of MDA-MB-231 cells without monoclonal antibody (control). Values indicate means ± SEM of results from three independent experiments. Significant differences were compared using one-way ANOVA; ** *P* < 0.01; *** *P* < 0.001

Chemokines released from cancer cells were reported to recruit Tregs [27, 29]. Using qRT-PCR and ELISA, we analyzed MDA-MB-231 and MDA-MB-468 cells treated with various concentrations of ZA to determine their expression of cytokines or other factors that affect Tregs function. The mRNA and protein expression of both CCL2 and CCL5 by IFN-γ activated breast cancer cells were significantly reduced in a dose-dependent manner by ZA treatment (Fig. 9). Our results imply that ZA treatment might reduce the ability of breast cancer cells to enhance the expansion and migration of Tregs by reducing their production of CCL2 and CCL5. To further confirm the role of MDA-MB-231 cell-secreted CCL2 and CCL5 as key factors that maintain Tregs migration, we measured the Tregs migration response to C.M. of MDA-MB-231 cells in the presence or absence of anti-CCL2 and anti-CCL5 antibodies. Figure 10 shows that either anti-CCL2 antibody or anti-CCL5 antibody significantly inhibited the Tregs migration induced by C.M. of MDA-MB-231 cells (*p* < 0.01). In addition, treatment with a combination of anti-CCL2 and anti-CCL5 antibodies nearly completely abolished the enhancement effect of MDA-MB-231 cell C.M. on Tregs migration (*p* < 0.001). The results demonstrated CCL2 and CCL5 expression was significantly increased when the cells treated with IFN-γ but reduced in a dose-dependent manner by ZA treatment.

## Discussion

Many studies have revealed that ZA has antitumor activity and might target the tumor microenvironment. Massaia et al found that ZA had multiple immune modulatory activities that allowed multiple myeloma dendritic cells to effectively handle the concurrent activation of cytotoxic γδ T-cells and MHC-restricted CD8^+^ αβ T-cells in vitro [22]. A transgenic breast cancer mouse model study showed that administration of ZA reduced the number of tumor-associated macrophages and caused macrophage repolarization [23]. Recently, Giannoni et al also reported that ZA impaired prostate cancer cell-induced polarization of M2-macrophages, reducing their pro-invasive effect on tumor cells and pro-angiogenic features [31]. ZA also reversed cancer-associated fibroblast activation by impairing the assembly of smooth muscle actin-α into fibers. These results revealed that ZA-induced stromal normalization impairs cancer-stromal cells crosstalk, resulting in the blockage of primary tumor growth and metastases [31]. In our previous report, we demonstrated that the migration of Tregs towards cancer cells was significantly inhibited after ZA treatment [26]. Our data also suggested that ZA inhibited the expansion and immunosuppressive function of Tregs in vitro. The ZA-mediated attenuation of immune evasion and tumor progression was mediated via inhibition of Tregs recruitment and expansion by the tumors. We also demonstrated that treatment with ZA significantly inhibits cancer cell migration by blocking the RANK/RANKL pathway induced by Tregs. This observation is consistent with previous studies showing that patients with breast cancer might benefit from Tregs depletion leading to a reduction in local immunosuppression and removal of a primary source of RANKL required for tumor metastasis [32, 33]. Our data on the effect of ZA on Tregs gene expression showed that the Tregs immunosuppressive function was suppressed by down-regulation of CTLA4, PD1, and RANKL. CTLA4 and PD-1 expression by Tregs is constitutive and critical to their immunosuppressive function [34–36]. The RANK-RANKL signaling pathway is critically involved in regulating the immunosuppressive function of Tregs [37], which can be modulated by ZA.

In this study, C.M. from MDA-MB-231 cells significantly enhanced the proliferation, migration, and immunosuppressive function of Tregs (Figs. 4, 5, and 6). In contrast, pretreatment of MDA-MB-231 cells with ZA significantly attenuated their ability to stimulate Treg proliferation, migration, and immunosuppressive function. ZA treatment significantly decreased IDO mRNA expression in triple negative breast cancer cells after IFN-γ treatment (Fig. 8). Because it is known that the level of IDO activity and the size of the immunosuppressive Tregs subpopulation vary in parallel [38], we conclude that ZA effectively inhibits breast cancer cells to induce expansion of Tregs at least partially via inhibition of the kynurenine/IDO axis. Ghigo et al also found similar effects of ZA, i.e., down-regulation of the IDO expression of malignant mesothelioma cells, facilitation of the proliferation of T-cells, and inhibition of the expansion of Tregs [38]. Additionally, MDA-MB-231 cell expressions of both chemokines CCL2 and CCL5, which are the major chemoattractants for Tregs, were significantly reduced by ZA treatment in a dose-dependent manner (Figs. 9). CCL2, CCL5 and IDO play key roles in promoting tumor growth and progression partially by inhibiting the immune response against cancer through Tregs. Therefore, anti-CC2 / CCL5 and IDO inhibitor could represent new strategies for cancer treatment [5, 10, 39]. Based on our present data and previous reports, ZA inhibited the interaction between Tregs and breast cancer cells and synergistically acted with cyotokine or IDO inhibitors to alert the tumor microenvironment and to enhanced anti-tumor immunity.

Holen et al recently reported that anti-tumor effects of ZA on breast cancer cell function differ according to their estrogen receptor (ER) status [40], suggesting that ZA’s effect on breast cancer cell line proliferation might depend on ER status. In their study, ZA significantly inhibited proliferation of ER-negative MDA-MB-231 cells but not ER-positive MCF-7 cells. We also found that C.M. of MDA-MB-231 cells (ER negative) significantly expanded Tregs number, induced Tregs chemotatic migration, and enhanced Tregs immunosuppressive activity. However, the C.M. of MCF-7 cells (ER positive) failed to support Tregs expansion and migration (Additional file 2: Figure S2 and Additional file 3: Figure S3). In addition, ZA appeared to have little effect on the interaction between MCF-7 cells and Tregs (data not shown). Clezardin et al [41] reported that IPP accumulation in ZA-treated breast cancer cells might be recognized by Vγ9Vδ2 T-cells as tumor phosphoantigens and induce Vγ9Vδ2 T-cell expansion, promote Vγ9Vδ2 T-cell chemotaxis to the tumor, and increase Vγ9Vδ2 T-cell cytotoxicity. IPP production differed markedly between different human breast cancer cell lines post-ZA treatment. Estrogen receptor positive breast cancer cell lines, such as MCF-7, produced higher IPP levels than estrogen receptor negative cell lines, such as MDA-MB-231. Their findings suggest that patients with estrogen-receptor-positive type breast cancer (with cells that produce higher IPP levels after ZA treatment) are most likely to benefit from Vγ9Vδ2 T-cell-mediated immunotherapy. Collectively, the data show that the effects of ZA treatment on the immune response to breast cancer depend on the hormonal receptor status of the cells.

## Conclusion

We showed that 1) breast cancer cells promote the expansion, chemotactic migration, and immunosuppressive function of Tregs and that 2) ZA inhibits the ability of breast cancer cells in a dose-dependent manner to promote Tregs proliferation, migration, and function at least partially due to the ZA-induced reduction of breast cancer cell expression of selected factors/chemokines (CCL2, CCL5, IDO, etc.). Our data suggest that ZA can modulate the interaction between Tregs and breast cancer cells. The efficacy of ZA was demonstrated to be a potential therapeutic approach to inhibit the aggressiveness cancer by blocking the supportive role of the tumor microenvironment in pathogenesis.

## Additional files

Additional file 1: **Figure S1.** C.M. of MDA-MB-231 cells enhanced Tregs proliferation. Total counts of viable Tregs stimulation in the presence of 100 U/ml rIL-2 and Dynabeads® Human Treg Expander with or without C.M. of MDA-MB-231 cells were calculated at the indicated times. Proliferation was expressed as the percentage of cell numbers relative to that at day 1 (100%). Data are representative of three independent experiments. The difference was compared using Student’s t-test. **, *P* < 0.01; *** *P* < 0.001. (JPG 75 kb)

Additional file 2: **Figure S2.** The effects of C.M. from MCF-7 cells on Tregs proliferation. Total counts of viable Tregs stimulation in the presence of 100 U/ml rIL-2 and Dynabeads® Human Treg Expander with or without MCF-7 cells C.M. at the indicated times were calculated. The proliferation was expressed as the percentage of the cell numbers relative to that at day 1 (100%). Data are representative of three independent experiments. (JPG 74 kb)

Additional file 3: **Figure S3.** The effect of C.M. from breast cancer cells on Tregs migration. Tregs (5 × 10 ^4^) were placed in the upper chambers. Migration of Tregs into the lower chambers containing DMEM with 2% FBS, C.M. of MCF-7 cells and MDA-MB-231 cells after 2 h was analyzed. The chemotaxis index shown compares migration with the response of Tregs to DMEM with 2% FBS. Values are means ± SEM of results from three independent experiments in duplicate. ***p < 0.001. (JPG 68 kb)

## Abbreviations

BP: Bisphosphonate
C.M.: Conditioned media harvested from MDA-MB-231 breast cancer cells consisting of DMEM
CCL2/MCP1: Chemokine C-C motif ligand 2
CCL5/RANTES: Chemokine C-C motif ligand 5
IDO: Indoleamine 2, 3-dioxygenase
IFN-γ: Interferon-gamma
PBMCs: Peripheral blood mononuclear cells
RANK: Receptor activator of nuclear factor kappa-B
RANKL: Receptor activator of nuclear factor kappa-B ligand
Tregs: Regulatory T-cells
ZA: Zoledronic acid
