# Supplementary figures and images for: Zoledronic acid blocks the interaction between breast cancer cells and regulatory T-cells
Source: BMC Cancer. 2019 Feb 26;19:176. doi: 10.1186/s12885-019-5379-9 (PMC6390606; doi:10.1186/s12885-019-5379-9)

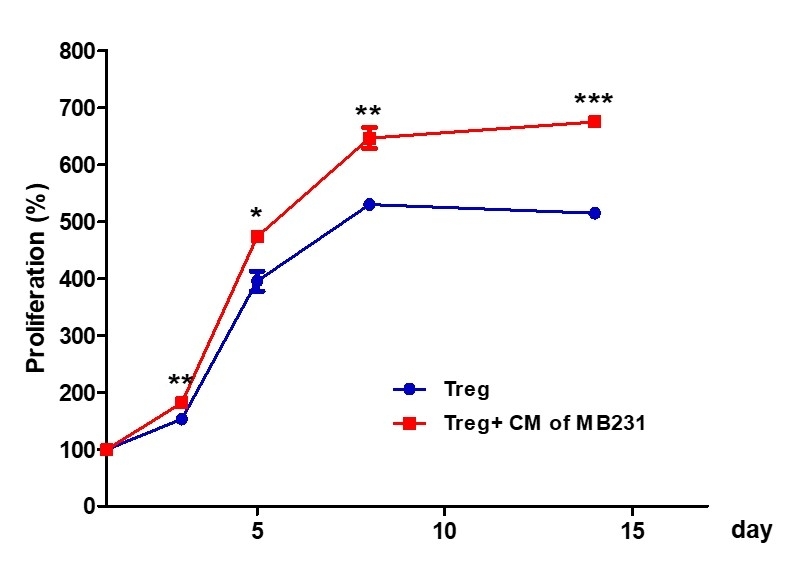

Supplement: Supplementary file 1 — Figure S1. C.M. of MDA-MB-231 cells enhanced Tregs proliferation. Total counts of viable Tregs stimulation in the presence of 100 U/ml rIL-2 and Dynabeads® Human Treg Expander with or without C.M. of MDA-MB-231 cells were calculated at the indicated times. Proliferation was expressed as the percentage of cell numbers relative to that at day 1 (100%). Data are representative of three independent experiments. The difference was compared using Student’s t-test. **, P < 0.01; *** P < 0.001. (JPG 75 kb) [file 12885_2019_5379_MOESM1_ESM.jpg]

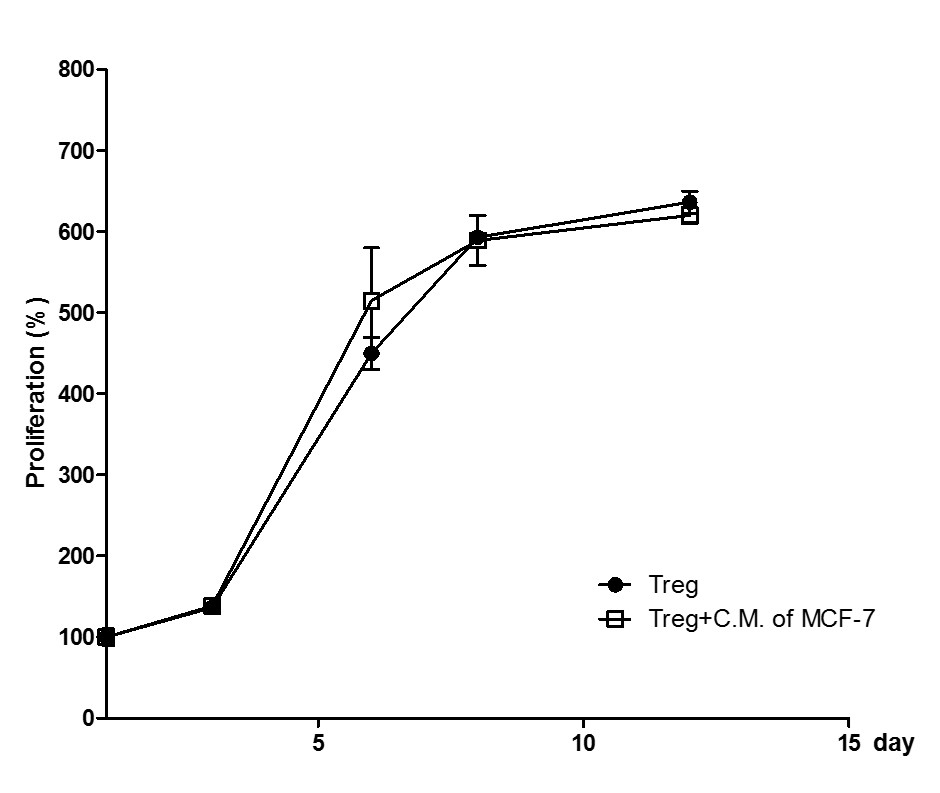

Supplement: Supplementary file 2 — Figure S2. The effects of C.M. from MCF-7 cells on Tregs proliferation. Total counts of viable Tregs stimulation in the presence of 100 U/ml rIL-2 and Dynabeads® Human Treg Expander with or without MCF-7 cells C.M. at the indicated times were calculated. The proliferation was expressed as the percentage of the cell numbers relative to that at day 1 (100%). Data are representative of three independent experiments. (JPG 74 kb) [file 12885_2019_5379_MOESM2_ESM.jpg]

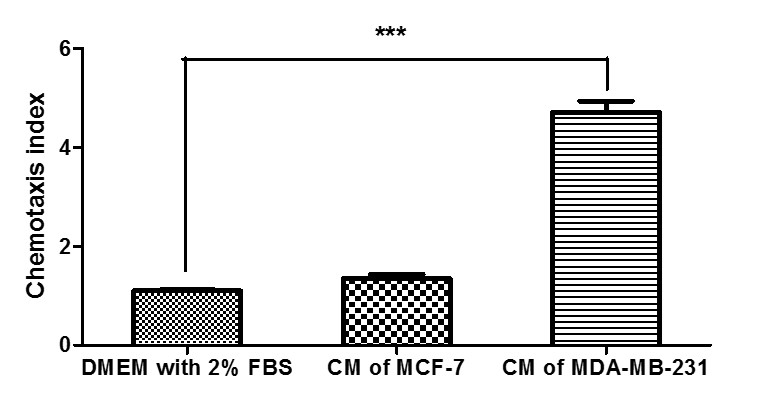

Supplement: Supplementary file 3 — Figure S3. The effect of C.M. from breast cancer cells on Tregs migration. Tregs (5 × 10 4) were placed in the upper chambers. Migration of Tregs into the lower chambers containing DMEM with 2% FBS, C.M. of MCF-7 cells and MDA-MB-231 cells after 2 h was analyzed. The chemotaxis index shown compares migration with the response of Tregs to DMEM with 2% FBS. Values are means ± SEM of results from three independent experiments in duplicate. ***p < 0.001. (JPG 68 kb) [file 12885_2019_5379_MOESM3_ESM.jpg]
